# Co-registration of glucose metabolism with positron emission tomography and vascularity with fluorescent diffuse optical tomography in mouse tumors

**DOI:** 10.1186/2191-219X-2-19

**Published:** 2012-05-07

**Authors:** Xiao Tong, Anikitos Garofalakis, Albertine Dubois, Raphaël Boisgard, Frédéric Ducongé, Régine Trébossen, Bertrand Tavitian

**Affiliations:** 1 CEA, Institut d’Imagerie Biomédicale (I2BM), Service Hospitalier Frédéric Joliot (SHFJ), Laboratoire d’Imagerie Moléculaire Expérimentale, 4 place du Général Leclerc, 91401, Orsay Cedex, France; 2 INSERM U1023, Université Paris Sud, 4 place du Général Leclerc, 91401, Orsay Cedex, France

**Keywords:** Co-registration, fDOT, PET, Fiducial marker detection, Optical surface image, Neuroendocrine tumors, MEN2A

## Abstract

**Background:**

Bimodal molecular imaging with fluorescence diffuse optical tomography (fDOT) and positron emission tomography (PET) has the capacity to provide multiple molecular information of mouse tumors. The objective of the present study is to co-register fDOT and PET molecular images of tumors in mice automatically.

**Methods:**

The coordinates of bimodal fiducial markers (FM) in regions of detection were automatically detected in planar optical images (*x*, *y* positions) in laser pattern optical surface images (*z* position) and in 3-D PET images. A transformation matrix was calculated from the coordinates of the FM in fDOT and in PET and applied in order to co-register images of mice bearing neuroendocrine tumors.

**Results:**

The method yielded accurate non-supervised co-registration of fDOT and PET images. The mean fiducial registration error was smaller than the respective voxel sizes for both modalities, allowing comparison of the distribution of contrast agents from both modalities in mice. Combined imaging depicting tumor metabolism with PET-[^18^ F]2-deoxy-2-fluoro-d-glucose and blood pool with fDOT demonstrated partial overlap of the two signals.

**Conclusions:**

This automatic method for co-registration of fDOT with PET and other modalities is efficient, simple and rapid, opening up multiplexing capacities for experimental *in vivo* molecular imaging.

## Background

The complexity of tumors and their sophisticated interactions with their environment call for imaging methods capable of detecting a diversity of tumor hallmarks [1,2]. Positron emission tomography (PET) with ^18^ F]2-deoxy-2-fluoro-d-glucose (FDG), the most efficient imaging method to detect cancer, is an indicator of tumor energy metabolism dominated by aerobic glycolysis in both cancer and tumor-associated inflammatory cells [3]. However, FDG-PET carries no information about other cancer hallmarks such as angiogenesis, replicative immortality, evasion of growth suppressors, capacity to metastasize, and yields at best indirect information on resistance to apoptosis and proliferation [1]. PET imaging with other radiotracers can complement FDG but cannot be performed in the same imaging session. It also increases radiation exposure. As far as experimental molecular imaging is concerned, multiple PET sessions are difficult to envision on a large scale because of high cost and low practicability.

Recent progress in fluorescence diffuse optical tomography (fDOT) has made 3-D optical molecular imaging of live animals a reality [4-9]. In contrast to imaging modalities demanding heavy instrumentation such as PET, fDOT is based on relatively simple hardware that does not require a sophisticated technological environment or tedious safety precautions. fDOT offers nano-molar sensitivity [5], long follow-up times (days) and is relatively low cost, and opens *in vivo* molecular imaging to a huge portfolio of fluorescent probes and beacons [5,6,8,10]. We [5] and others [8] have proposed the combination of PET-FDG and fDOT as a method of choice to provide multiple molecular data on experimental tumors in mice.

Given the small size (50–500 mm^3^) of tumors in mice and the resolution of small-animal PET and fDOT scanners (1–2 mm), accurate and reliable co-registration between both modalities is essential. Among different co-registration methods that have been developed, such as geometrical co-calibration [11] and dynamic contrast methods [12], the use of fiducial markers (FM) [8] in close position to the body of the animal is the most straightforward and universal approach today. The coordinates of the FM in images acquired independently is used for the geometrical transformations leading to the fusion of images. Co-registration of large data sets from different imaging modalities results in time-consuming, tedious and operator-dependant image analyses when performed manually. Therefore, methods for the automatic identification of the FM's coordinates have been developed for co-registration of computed tomography (CT), PET and magnetic resonance imaging (MRI) modalities [13-18]. However, so far, these methods have not been adapted to co-registration with fDOT because fDOT reconstructions are spatially restricted and do not cover the FM positioning.

Here, we introduce the use of surface images obtained during the fDOT acquisition session for the automatic identification of the FM's positions in space. Surface reconstruction by laser patterning [19] can retrieve the 3-D surface of the subject and of the FM in close vicinity. Surface reconstruction is directly implemented into the 3-D fDOT-PET combined image. We show that this method efficiently performs co-registration of fDOT and PET images of the same mouse with a co-registration error (fiducial registration error, FRE) smaller than the intrinsic resolution of PET and fDOT. This new automatic method facilitates the accurate co-registration of fDOT with PET and other imaging modalities. As a proof of concept, we imaged mice bearing neuroendocrine tumors for glycolytic metabolism with FDG-PET and for tumoral blood pool with a fluorescent blood pool contrast agent. We show that these two tumoral hallmarks occupy partially overlapping volumes, suggesting that the tumor-induced induction of blood supply could be spatially restricted to a portion of the tumor mass.

## Materials and methods

### Plexiglas cubes containing FM

**Figure 1:**
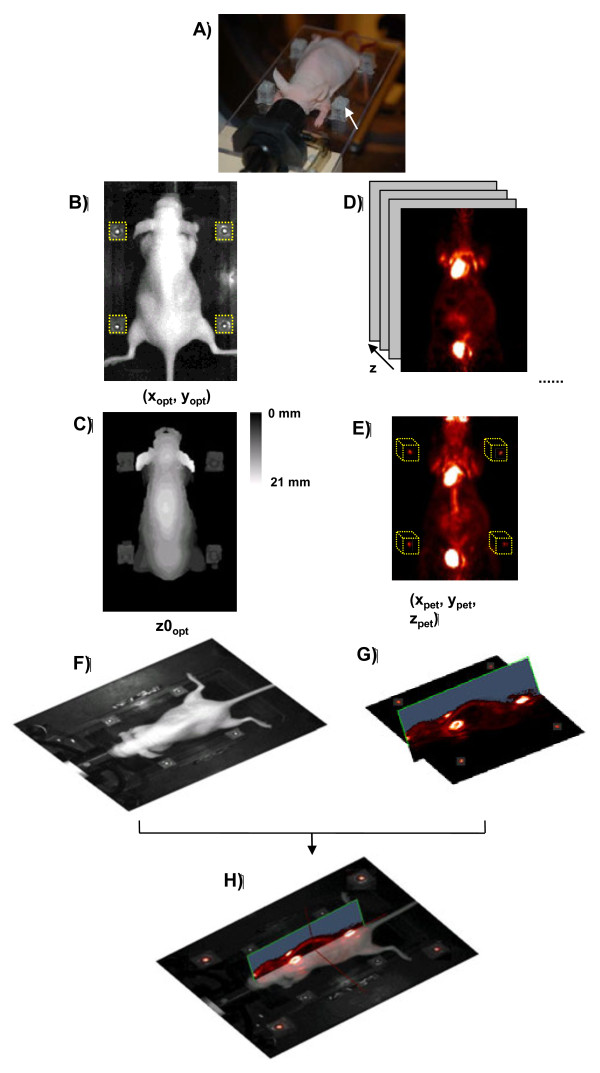
**Outline of the automatic co-registration method for optical and micro-PET images**. **(A)** View of the multimodality mouse support system showing the four Plexiglas cubes containing the fiducial markers (FM). **(B)** Planar white light image of the subject used for the extraction of the planar coordinates of the FM. **(C)** Optical surface image used for the extraction of the *z* (vertical) coordinates of the FM (*z*0_opt_). **(D)** A stack of coronal PET slices that correspond to the different *z* positions. **(E)** Projection image of the PET volume signal. The localization of the FM where their coordinates correspond to the radiation signal (*x*_PET_, *y*_PET_, *z*_PET_) is highlighted by dotted yellow rectangles. **(F)** Bird's eye view of the optical image of the mouse. **(G)** Bird's eye view of the micro-PET volume image. **(H)** Assembled image of the co-registered optical 3-D PET images. The image shows the coronal view of the animal with signals from both modalities, while the sagittal view depicts only the PET signal. Four zoom images of the co-registered FM are shown at the four corners of the image.

The FM were composed of four sources of germanium-68 (74 kBq; diameter, 1 mm; and length, 0.5 mm; Isotop Product Laboratories, Valencia, CA, USA) originally designed for PET-CT co-registration, included in the center of four Plexiglas cubes of 1 × 1 × 1 cm. A spot of 2 mm diameter was drawn with standard white liquid corrector (Tipp-Ex®, Bic, Clichy, France) on top of each Plexiglas cube, exactly above the ^68^Ge sources. The cubes were then glued permanently to a custom made transparent Plexiglas mouse supporting plate at four corners close to the position of the mouse on the plate (Figure 1A).

### Animal experiments

Animal experiments were performed under an animal use and care protocol approved by the animal ethics committee and conducted in accordance with Directive 2010/63/EU of the European Parliament. Six female nude mice (body weight of approximately 25 g) were obtained from Elevage Janvier (Le Genest Saint Isle, France) and received a subcutaneous injection in the flank of 10^6^ PC12-multiple endocrine neoplasis syndrome type 2A (MEN2A) cells [20]. The mice were anesthetized by continuous gaseous anesthesia (1–2% isoflurane in O_2_) and imaged sequentially by fDOT and PET. The near-infrared (NIR) fluorescent dye Sentidye® (20 nmol; Fluoptics, Grenoble, France) was injected 3 h before starting the fDOT acquisition at a volume of 100 μL. FDG (7,400 kBq in 100 μL; Flucis, IBA, France) was administered 1 h before the PET scan. Each mouse underwent a 20-min fDOT acquisition followed by a 30-min PET acquisition. The anesthetized mice were transferred from the fDOT to PET scanner by means of the mouse supporting plate, while great care was taken to avoid movement of the animal in regard to its support. The contact of the ventral side of a nude mouse with the Plexiglas surface of the mouse holder is sticky enough to ensure that the mouse is not moving when transferred between scanners located at the same room.

### Acquisition of the optical images

**Figure 2:**
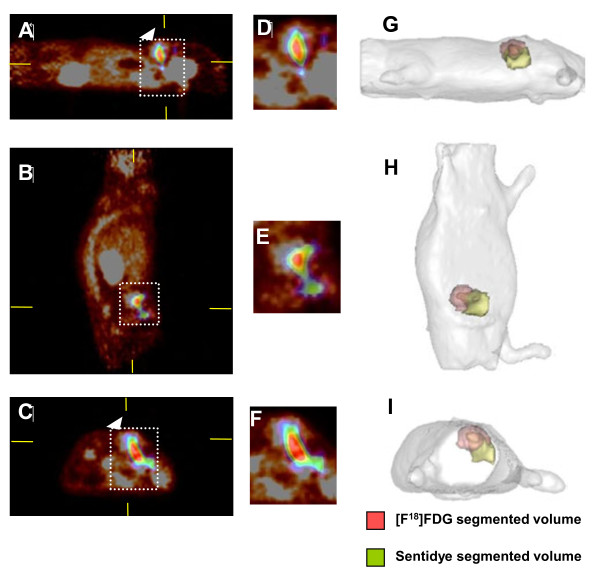
**Co-registration of fDOT and micro-PET in a mouse bearing a multiple endocrine neoplasis syndrome type 2A tumor xenograft**. Left images are the fusion fDOT/PET image in the sagittal **(A)**, coronal **(B)** and axial **(C)** planes. The corresponding zooming images focus on tumor region the in the sagittal **(D)**, coronal **(E)** and axial **(F)** planes. Color scales are ‘temperature’ (dark red to brilliant yellow) for PET and ‘rainbow’ (blue to white) for fDOT. The white-dotted rectangles point at the reconstruction mesh of the fDOT, while the white arrows indicate the position of the tumor. Right images are the PET and fDOT signals rendered to the envelope of the mouse corresponding to the sagittal **(G)**, coronal **(H)** and axial **(I)** projections. Pink volume, [^18^ F]FDG segmented volume inside the tumor; yellow volume, Sentidye® segmented volume inside the tumor. Both volumes were extracted from the volumes of interests used for the quantification of each type of signal.

Images were acquired in the 3-D optical imager TomoFluo3-D (Cyberstar, Grenoble, France) [7]. To obtain the position of the FM, two optical images covering both the subject and the FM were acquired with the mouse placed in prone position: (1) a planar white light image recorded from a camera snapshot yielding the *x* and *y* coordinates (Figure 1B), and (2) an image of the 3-D surface of the animal, acquired by rapid consecutive camera shootings during axial scanning with an inclined green planar laser of the TomoFluo3-D, yielding the *z* coordinates (Figure 1C). One image was recorded for each laser position during laser scanning of the animal in the axial direction, and all images were then combined into a single image [7]. A surface image of the animal was then reconstructed from the intersection curve between the surface of the animal and the surface of the supporting plate by triangulation [7].

The fDOT image was obtained by a 20-min scan of a defined volume of interest covering the tumor using excitation by a 680-nm laser on the anterior side of the animal [7] and recording with a CCD camera fixed above its dorsal side. The scanning grid consisted of 7 × 6 sources in steps of 2 mm, and the detection area was 15 × 13 mm^2^. A 2 × 2 binning was applied, and the mesh volume corresponding to the detection area was mathematically discretized in voxels of 0.67 × 0.67 × 1 mm^3^ size to build the reconstruction mesh volume. Finally, the inverse problem of the tomographic reconstruction was solved with the algebraic reconstruction technique [7].

### PET image acquisition

A 30-min scan was acquired in a Focus 220 MicroPET scanner (Siemens, Knoxville, TN, USA). Image acquisition and reconstruction used the MicroPET Manager Software (Siemens-Concorde Microsystems) based on a filtered back-projection algorithm. The attenuation correction was based on the segmentation of the emission map [21]; the dimensions of reconstruction volumes were 256 × 256 × 95 with a voxel size of 0.475 × 0.475 × 0.796 mm^3^. The counts were decay-corrected and expressed in Bq/cm^3^.

### Image co-registration

As the three optical images (white light image, surface image and fDOT reconstruction) were acquired in the same spatial referential, the transformation matrix between the fDOT image and the white light image *T*_*fDOT*−*photo*_ is calculated using the intrinsic parameters of fDOT. Hence, the *T*_*fDOT*-*PET*_ transformation matrix for fDOT to PET co-registration is a product of *T*_*fDOT*-*photo*_ and *T*_*photo*-*PET*_:

(1) $$T_{fDOT-PET}=T_{fDOT-photo}\times T_{photo-PET}$$

The co-registration (*T*_*photo-PET*_) method was processed in four steps:

1. Detection of the optical planar (*x*, *y*) FM coordinates.

The Tipp-Ex® spot drawn on the top of each Plexiglas cube helped visualize the planar position of the FM in optical images. Four square-shaped regions of detection (ROD) were assigned onto predetermined positions in the planar white light image (Figure 1B). Each ROD had a size of 6 × 6 mm, corresponding to 30 × 30 pixels in the concatenated mouse photograph, i.e., three times larger than the Tipp-Ex® spots' dimensions in order to obey the Nyquist-Shannon sampling theorem [22] while avoiding parasite signals from the mouse body. The first step consisted in the automatic detection of the *x* and *y* coordinates of the FM based on the maximal intensity inside the corresponding RODs (Figure 1B). Three image preprocessing steps were then performed successively: (1) filtering with a 3 × 3 median filter that eliminated most of the noise present in the RODs, (2) high-pass thresholding of image pixel intensities at a threshold value of 90 % of the maximum intensity and (3) application of a Deriche's recursive Gaussian filter [23] in order to center the gradient intensity change in the images of the Tipp-Ex® spots. Following these three steps, the coordinates of the local maximum intensity in each ROD coincided with the center of the FM signal given by the Tipp-Ex® spots in the planar white light image and assigned positions (*x*_opt_, *y*_opt_) of the FM.

2. Detection of the optical altitude (*z*) FM coordinates.

Since the optical surface image (Figure 1C) and the planar white light image are concatenated in the same orientation and the same pixel size, the (*x*, *y*) coordinates in both images correspond directly. The intensity values of the optical surface image representing the distance between the upper surface of the FM and the supporting plate were measured at position *x*_opt_, *y*_opt_ to yield the *z*0_opt_ value of the upper surface of the FM. Altogether, the combination of the optical surface image and the planar white light image allowed assigning full 3-D coordinates (*x*_opt_, *y*_opt_, *z*0_opt_) to each FM in the optical image.

3. Detection of PET FM coordinates.

Four 3-D RODs of 9 mm^3^ (dimensions three times larger than the dimension of FM signal in PET image) were defined in the PET volume image (Figure 1E). Following completion of the same image preprocessing steps as for the detection of the optical planar coordinates, the local maximum was detected in each ROD to yield the coordinates (*x*_PET_, *y*_PET_, *z*_PET_) of the FM in the PET volume image. Since the optical and PET signals from the FM do not coincide in the *z* dimension (i.e., the optical signal is on top of the Plexiglas cube, and the PET signal inserted inside the cube), a distance *dz* was added to account for translation in the *z* direction after the calculation of the rigid transformation matrix between the optical and PET image.

4. Transformation from the mouse photograph to the PET volume.

A rigid transformation with translation and rotation was applied to co-register the optical and PET coordinates of the FM. With *Po* = {*Po*_1_, *Po*_2_, *Po*_3_, *Po*_4_} and *Pp* = {*Pp*_1_, *Pp*_2_, *Pp*_3_, *Pp*_4_} being the four FM positions in the optical and the PET volume images, respectively, the translation *T* and rotation *R* were defined as Equation 2:

(2) Pp=R∗Po+T

The algorithm to compute the transformation *R*, *T* used the singular value decomposition (SVD) [24] approach to find the least square error criterion (Equation 3),

(3) $$\sum_{i=1}^{N}E=\sum_{i=1}^{N}{\left|\left|Ppi-\left(RPoi+T\right)\right|\right|}^{2}\text{,}\;\left(\text{where }N=4\text{ is the number of FM}\text{.}\right)$$

The point sets {*Pp*_*i*_} and {*Po*_*i*_} were imposed the same centroid for calculating rotation:

(4) $$\begin{array}{l} \bar{Pp}=\frac{1}{N}\sum_{i=1}^{N}Pp_{i}\;\hat{P}p_{i}=Pp_{i}-\bar{Pp}\\ \bar{Po}=\frac{1}{N}\sum_{i=1}^{N}Po_{i}\;\hat{P}o_{i}=Po_{i}-\bar{Po} \end{array}$$

Rewriting and reducing Equation 3:

(5) $$\begin{array}{c} \;\sum_{i=1}^{n}E=\sum_{i=1}^{N}{\left|\left|\hat{P}p_{i}-\hat{R}\;\hat{P}o_{i}\right|\right|}^{2}\\ =\sum_{i=1}^{N}\left(\hat{P}p_{i}^{T}\;\hat{P}p_{i}+\hat{P}o_{i}^{T}\;\hat{P}o_{i}-2\hat{P}p_{i}^{T}\;\hat{R}\;\hat{P}o_{i}\right) \end{array}$$

Transformation was expressed as Equation 6:

(6) $$\hat{T}=\bar{Pp}-\hat{R}\;\bar{Po}$$

The $\hat{R}$, $\hat{T}$ is the optimal transformation that maps the set {*Pp*_*i*_} to the {*Po*_*i*_}. Equation 5, also known as the orthogonal Procrustes problem [24,25], is minimal when the last term is maximal.

The optical images, being proportionally larger than the PET image due (1) to the difference in the pixel size between the optical planar image (0.21 × 0.21 mm) and the PET image (0.47 × 0.47 mm), and (2) to the parallax induced by the camera detecting the Tipp-Ex® spots from the upper surface of the cube, a scaling factor was applied by calculating the average distance of the points in the *x* and *y* axes between the two modalities as (*do*_*x*_*, do*_*y*_) and (*dp*_*x*_*, dp*_*y*_):

(7) $$\begin{array}{c} do_{x}=\left[\left(Po_{2}(x)-Po_{1}(x)\right)+\left(Po_{4}(x)-Po_{3}(x)\right)\right]/2\\ dp_{x}=\left[\left(Pp_{2}(x)-Pp_{1}(x)\right)+\left(Pp_{4}(x)-Pp_{3}(x)\right)\right]/2\\ do_{y}=\left[\left(Po_{3}(y)-Po_{1}(y)\right)+\left(Po_{4}(y)-Po_{2}(y)\right)\right]/2\\ dp_{x}=\left[\left(Pp_{3}(x)-Pp_{1}(x)\right)+\left(Pp_{4}(x)-Pp_{2}(x)\right)\right]/2 \end{array}$$

A 2 × 2 scaling matrix was then built:

(8) $$S=\left[\begin{array}{ccc} S_{x} & 0 & 0\\ 0 & S_{y} & 0\\ 0 & 0 & s_{z} \end{array}\right]$$

where $Sx=\frac{do_{x}}{dp_{x}}$, $Sy=\frac{do_{y}}{dp_{y}}$ and *S z* = 1 (i.e., no scaling in the *z* direction).

After correcting for distance *dz*, the final transformation matrix *T*_*photo-PET*_ for the optical photograph to the PET volume was:

(9) $$T_{photo–PET}=\left[\begin{array}{cccc} \hat{R}S_{11} & \hat{R}S_{12} & \hat{R}S_{13} & \hat{T}x\\ \hat{R}S_{21} & \hat{R}S_{22} & \hat{R}S_{23} & \hat{T}y\\ \hat{R}S_{31} & \hat{R}S_{32} & \hat{R}S_{33} & \hat{T}z \end{array}\right]$$

where *RS* are the rotation matrix elements *R* multiplied by the scaling matrix elements *S* of Equation 8.

Applying the matrix in Equation 9 to the optical photography (Figure 1F) aligned the 3-D PET image (Figure 1G) to yield the co-registered bimodal image shown in Figure 1H.

#### *The barycenter-based method*

The barycenter-based method [13-18] calculates average location of body's mass weighted in space. The barycenter was calculated as the center of mass *B* of a system (i.e., all voxels within the RODs) defined as the average of their positions *ri*, weighted by their mass *mi*:

(10) $$B=\frac{\sum m_{i}r_{i}}{\sum m_{i}}$$

For the mouse photograph, as the barycenter was calculated in two dimensions, the left upper corner of each ROD was taken as origin, and the vector *r*_*i*_ corresponded to the relative position from this origin to each pixel within the ROD. The signal intensity of each pixel inside the ROD gave the value of *m*_*i*_. For the PET image, the barycenter was calculated in 3-D, and the position of the first voxel (left upper in coronal view and first section in the *z* direction) of the 3-D ROD was taken as the origin. The position of each voxel relative to this point was taken as *r*_*i*_ and the value of each voxel as *m*_*i*_. The barycenter calculated for each ROD represented the FM's position.

## Results

### Co-registration procedure

The general outline of the co-registration method is depicted schematically in Figure 1. Five steps are implemented successively: (1) registration of (*x*_opt_, *y*_opt_, *z*0_opt_), i.e., the *x*, *y* and *z* coordinates of the optical FM using both the white light (Figure 1 B) and optical surface (Figure 1C) images; (2) calculation of *T*_*fDOT*-*photo*_, the transformation matrix from the fDOT reconstruction image to the whole body image; (3) registration of (*x*_PET_, *y*_PET_, *z*_PET_), i.e., the *x*, *y* and *z* coordinates of the FM in the PET images (Figure 1D,E); (4) calculation of *T*_*photo*-*PET*_, the transformation matrix from optical to PET volumes (Figure 1H); and (5) calculation of *T*_*fDOT*-*PET*_, the transformation matrix for the co-registration of the fDOT volumes with the PET volumes.

The complete image processing method was written in C++ and integrated in Python with a user-friendly interface within the Brainvisa image processing software (http://brainvisa.info/index_f.html) [26]. Input files include (1) the mouse photograph, (2) the optical surface scan, (3) a header file containing the position of the fDOT image relative to the camera's field of view, and (4) the PET volume image. With this user-friendly toolbox, three transformation matrices *(T*_*fDOT-photo*_, *T*_*photo-PET*_, and *T*_*fDOT-PET*_*)* are calculated and sent as outputs. The program is completed in 20–30 s on a Dell Precision^TM^ T7500 (Dell SA, Montpellier, France) workstation with a 64-bit Intel® Xeon® quad-core processor (Intel Corporation, Montpellier SAS, France).

### Quantitative evaluation of the co-registration

#### Fiducial registration error-planar justification

To evaluate quantitatively the performance of our co-registration method, we calculated the FRE [13,15,16] as the root mean square (rms) distance of the positions of the FM in the two image modalities after co-registration in six independent experiments in six mice. The resulting FRE values are the mean FRE calculated in four points of the FM for each mouse. We compared the FRE of our maximum intensity (MI) method with that of the manual co-registration (MC) as a reference method and of the barycenter-based (BC) method [13-18]. The errors were calculated in the co-registered images with the distance in pixel unit multiplied by the pixel size of each modality.

(11) $$FRE_{opt-PET}\left(mm\right)=\{\begin{array}{c} \Delta pixel_{\mathrm{opt}}\times pixelsize_{\mathrm{opt}}\\ \Delta pixel_{\mathrm{PET}}\times pixelsize_{\mathrm{PET}} \end{array}$$

Due to the differences of the pixel size in the fDOT image and the PET image, individual FRE values are calculated for each modality. Using the BC approach, the FRE was 0.55 ± 0.11 mm (mean ± standard deviation; *n* = 6) and 0.45 ± 0.08 mm for optical and PET images, respectively. Using the MI approach, the FRE was 0.26 ± 0.06 mm and 0.25 ± 0.12 mm for optical and PET images, respectively. The FRE of the MC approach was 0.28 mm ± 0.05 mm in optical images and 0.22 ± 0.09 mm for PET images. Comparison of the three approaches (Table 1) showed that the MI approach produced average FRE smaller than the BC approach and in the same range as that of the MC approach.

**Table 1:** Fiducial registration errors

**FRE**		**MC (mm)**	**MI (mm)**	**BC (mm)**
Optical	Mean	0.279	0.259	0.545
	SD	0.05	0.06	0.11
PET	Mean	0.217	0.256	0.448
	SD	0.09	0.12	0.08

*MC* manual co-registration, *MI* maximal intensity co-registration, *BC* barycenter-based co-registration, *SD*, standard deviation of the mean (*n* = 6 for all calculations).

In all cases, statistically significant differences were found between the FRE values of the MI and BC approaches (student's *t* test, *p* = 0.0003 and *p* = 0.0007 for optical and PET images, respectively), while no statistically significant differences were found between the MI and the MC approach (*p* = 0.51 and *p* = 0.55 for optical and PET images, respectively). Taken together, these results indicate that the MI approach has the same co-registration performance with the MC approach and that both are more precise than the BC approach.

#### Evaluation of co-registration quality in the z direction

In order to evaluate the co-registration error in the *z* direction of our MI approach, the mouse surface image was fused with the PET image using the transformation matrix *T*_*photo-PET*_. The average error in the *z* direction was 0.37 ± 0.06 mm, a value smaller than the voxel size in the *z* direction (1 mm in optical images and 0.475 mm in PET images). Taking into account the error in the *z* direction, the 3-D FRE value of our MI approach was 0.452 mm for the optical image and 0.446 mm for the PET image.

#### Fiducial localization error and target registration error

The fiducial localization error (FLE) represents the rms error distance between the estimated and the true positions of a FM [13,15]. Here, it was calculated following Fitzpatrick et al. [15]:

(12) $${〈FRE〉}^{2}=\left(1-\frac{2}{N}\right){〈FLE〉}^{2}$$

Using the average value of the FRE calculated previously for MI approach (here, *N* = 4), the FLE was 0.35 mm for the PET images and 0.37 mm for the optical images. The FLE values were smaller than the 3-D FRE values that are independent from the fiducial configuration [15].

The target registration error (TRE) is defined as the distance after co-registration between the corresponding points in two modalities that not have been used for calculating the co-registration. TRE is derived from the mean FRE and FLE [15]:

(13) $${〈TRE〉}^{2}={〈FLE〉}^{2}-{〈FRE〉}^{2}$$

The mean TRE was 0.26 mm for optical images and 0.25 mm for PET image. The calculated TRE was similar as the FRE for both modalities, indicating that the co-registration error derived from the FM position and other positions on the image remain coherent.

### Co-registration of tumor vascularity and metabolism

The fDOT-to-PET co-registration method was applied to six female nude mice bearing xenografts tumor of PC12-MEN2A cancer cells that mimic a human medullar thyroid carcinoma [20]. The diameter of tumors ranged from 4.5–8 mm, corresponding to tumor weights of 50–270 mg. Each mouse received two injections; the first injection was the fluorescent probe Sentidye®, a probe that passively accumulates in tumors by virtue of the enhanced permeability and retention effect, and the second one is FDG. Each mouse was imaged sequentially with fDOT and PET.

There was a strong correlation between the volume of the tumor and the total uptake of FDG, but no correlation between the tumor volume and the concentration of FDG in the tumor expressed in percent of injected dose per cubic centimeter. In other words, the radioactivity concentration remained independent of the tumor volume. The fDOT signal also increased with tumor size but plateaued for tumors larger than 150 mg, corresponding to a tumor with a diameter larger than 6 mm. Again, no correlation was found between tumor volume and the concentration of the fDOT signal in the tumor area.

The fDOT-PET fused images (Figure 2) showed the localization of the optical probe with respect to the glucose consumption of the tumor. After the RODs had been defined for the mouse holder as indicated above, fDOT and PET images were co-registered automatically and the localization of FDG and Sentidye® uptakes were compared directly. Tumor volumes measured based on PET-FDG uptake ranged from 53–271 mm^3^ (mean = 143, SD = 105); tumor volumes measured from fDOT after Sentidye® uptake ranged from 83–265 mm^3^ (mean = 170, SD = 86). There was no correlation between the volumes measured with the two modalities, i.e., the ratio of fDOT-based to PET-based tumor volumes varied over almost one order of magnitude (range: 0.35–3.1; mean = 1.6; SD = 1.0). More interestingly, co-registration of fDOT with PET showed that the coefficient of overlap between vascular accumulation of Sentidye®, and tumor uptake of FDG was 42 ± 14%, indicating that only part of the tumor was hypervascular while the majority of the fDOT signal appeared located in the vasculature surrounding the tumor (Figure 2D–F).

## Discussion

There is a clear trend towards multi-modal imaging of tumor as the best way to provide relevant information on a significant number of cancer hallmarks [1,27]. Recent studies have demonstrated the possibility to combine fDOT and PET imaging for co-registered localization of two or more biological processes at the molecular level [5,8]. These studies required the use of CT for the anatomical structure, therefore, leading to irradiation of the animal that has an effect on tumor cell development. This prompted us to develop direct co-registration of PET/fDOT without use of CT.

The method for co-registration of PET and fDOT presented here is based on the detection of the position coordinates of FM visible with both modalities. While detection of radiolabeled FM with PET is relatively straightforward, it is not trivial for fDOT since planar optical images do not provide any information on the position of the FM in the *z* axis. Moreover, the acquisition of 3-D fDOT data is slow and would require extremely long acquisition times to scan a complete volume of the mouse body. The FM is not contiguous to the mouse body, requiring simultaneous 3-D acquisition for unconnected objects (i.e., mouse body plus FM), a difficult task to implement. Finally, mice need complete depilation since hair and fur skew the optical transmission of fluorescent signals. With these limitations in mind, we developed a method based on the combination of a planar optical image with a surface scan of the animal that automatically determine the full 3-D coordinates of four FM placed around the body of the animal. Interestingly, the planar image and the surface scan are acquired in the same referential as the fDOT volume and, thus, intrinsically co-registered. After an initialization step to define the regions in which the FM should be detected (the RODs), this method allows for automatic detection of the coordinates of the FM and immediately co-registers the fDOT image with the PET image. Since the FM are permanently attached to the animal plate holder, the definition of the RODs needs to be performed only once for a given animal supporting plate and remains valid for all following imaging sessions with that plate.

The performance of co-registration with the present automatic method was comparable to that of manual methods and found to be better than that of the BC approach, supporting the view that our automatic co-registration method is at least as accurate as an experienced human observer. The method is based on a simple principle which is the same as the manual co-registration, i.e., detection of the points with maximum intensity, while barycenter approaches use intensity weighting. However, Wang et al. have reported that co-registration based on barycenter intensity weighting performed better than an unweighted intensity approach [13,14]. This apparent discrepancy is likely due to the shape of the signal from the FM in the PET images. The FM signal in PET is generally not well-rounded because of the noise generated by the reconstruction from punctiform sources known as the ‘star’ artifact. This adds irrelevant weight to the RODs and renders the detection of the barycenter largely dependent on the size and localization of the ROD leading to unpredictable and often poorly reproducible calculation of the FM's position. In contrast, detection by maximal intensity is well adapted to automatic co-registration and can be generalized to any ROD to give reliable co-registered image results. As long as the signals from the FM remain above the surrounding background in the defined regions, the size and shape of the RODs have no influence on the results and can be larger or smaller than three times the diameter of the FM signal size. The aforementioned evaluation results show the advantages of our method in terms of reliability and robustness. Moreover, the method allows the precise and fast co-registration of individually taken images without any operator-dependent bias which can be advantageous when a study involves a large amount of experiments.

Two general solutions are available to compute the point-based rigid body transformation *R*, *T* between the optical and PET coordinates: the iterative method [28] and the closed form solution method [24,29]. The closed form solution method is generally superior to the iterative method in terms of efficiency and robustness [24]. Eggert et al. have compared four popular algorithms [24], each of which compute the translational and rotational components of the transform in closed form, as the solution to a least squares error criterion given by Equation 3. They found the SVD to give the best results in terms of 3-D accuracy and stability [24]. This algorithm is, therefore, chosen in the present study.

The proposed co-registration method can easily be customized for specific applications. The method can also be applied to co-registration between fDOT and modalities other than PET, such as CT or MRI, as long as the images contain the relevant FM. In addition, the method is adaptable to most fDOT or planar optical imaging instruments with surface reconstruction. Moreover, once the RODs have been initialized for a mouse support with FM, multiple experiments can be co-registered automatically with the same or different mice. It is noteworthy that co-registration of fDOT and PET offers the interesting possibility to co-register and, therefore, visualize two purely molecular contrast-based imaging methods without the need for referring to another anatomical imaging method. Where FDG is routinely used for the staging of tumors, it can be complemented with fDOT optical images of a second tumor-related activity fused to the FDG readouts.

As a proof of concept, we co-registered images of two well-established hallmarks of cancer: deregulation of glucose consumption through aerobic glycolysis and increased vascularization through angiogenesis and neo-vessel formation. Automatic co-registration of glycolysis with FDG-PET and vascularity with Sentidye® fDOT clearly demonstrated that the two signals were independently distributed in multiple endocrine neoplasis syndrome type 2A (MEN2A-induced medullary thyroid carcinoma (MTC) xenografts). MEN2A syndromes are linked to a mutation of the rearranged during transfection (RET) proto-oncogene coding for a membrane tyrosine kinase receptor. The mutation induces constitutive activation of the RET signaling pathway through dimerization of RET and is found in families with hereditary MTC and other disorders [30]. There is evidence that RET constitutively activates angiogenesis, likely through increased VEGF secretion, in MTC [31], and RET inhibitors induce inhibition of angiogenesis [32]. The new blood vessels produced within tumors by chronically activated angiogenesis (‘angiogenic switch’) are abnormal in their structure, distorted, enlarged, leaky and cause erratic blood flow and hemorrhagic lakes within the tumor [33]. Sentidye® is a NIR fluorescence lipidic nano-particle that accumulates passively in the leaky, abnormal vascular network of MEN2A-MTC tumors. The observation that it distributes in a pattern distinct from that of FDG is indicative of the fact that the tumors are organized into regions with distinct underlying physiological and molecular characteristics. Assuming that the distribution of the FDG signal indicates the localization of cancer cells and serves as a reference to the optical signal [5], it can be concluded that the hypervascular part of the tumor covers approximately 40 % of the tumor area. The spatial accuracy of both PET and fDOT reconstructions has been validated with the use of CT in a previous study [5]. In addition, in a different set of combined PET/fDOT scans, the tumor area was sampled after sacrifice of the mice and sectioned for histology (data not shown). The *ex vivo* distribution of the probes matched the *in vivo* reconstructions perfectly. We, therefore, conclude that the mismatch in the observed contrast distribution between fDOT and PET is due to the nature of the process underlying probe distribution and not to artifacts in the co-registration method. Interestingly, high FDG uptake which reflects aerobic glycolysis is not superimposed with high vascularity in MEN2A-MTC tumors. It would be interesting to explore further the fine architecture of tumors with other molecular tracers in order to segment tumoral regions based on, e.g., oxygen pressure, pH, apoptosis, proliferation or other important hallmarks of cancer [1]. Alternatively, it could be envisioned to co-register the distribution of a labeled drug or refine the anatomical information or superimpose maps of diffusion or viscoelastic properties using other imaging techniques such as MRI or ultrasound.

In summary, co-registered fDOT-PET is a translational *in vivo* molecular imaging modality of simple implementation that brings relevant information to experimental studies of tumors. Accurate co-registration combining these two molecular imaging modalities likely to (1) facilitate *in vitro* to *in vivo* correlations through *ex vivo* fluorescent imaging of pathological samples [8], (2) document the mechanism of uptake of clinically used radiotracers and contrast agents by adding complementary molecular information [8], (3) decipher the changes induced by administration of therapy [9], and (4) validate the *in vivo* targeting capacity of new molecular probes prior to radioactive labeling for PET or SPECT or tagging with paramagnetic atoms for MRI [8]. Future applications could include the use of other types of fluorescent probes, in particular, those that are activated only after interaction with the target for the monitoring of a variety of tumor-related molecules [8]. Additionally, since optical imaging allows the imaging of several probes with distinct emission spectra at the same time, the concept of complementing the FDG signal with a growing number of information could be further extended. The benefits from the fusion of fDOT and PET in combination with CT are expected to give rise to scanners where the two modalities are integrated within the same apparatus, and there are ongoing efforts for the development of this type of methods [34-36]. The combination of these two modalities offers new opportunities for describing tissue physiopathology non-invasively at refined molecular levels and opens experimental molecular imaging to simultaneous detection of multiple molecular targets and activities (‘multiplexing’).

## Authors' contributions

XT carried out the automatic co-registration method, the programming in C++ and performed the statistical analyses, as well as contributed to the draft of the manuscript. AG carried out the animal (mouse) experiments and the fDOT reconstructions, and contributed to the draft of the manuscript and the design of the study. AD carried out the manual co-registration method and contributed to the draft of the manuscript. RB participated in the design of the study and carried out the PET acquisitions. FD contributed to the mouse experiments and provided comments on the manuscript. RT participated in the design of the study. BT conceived of the project and participated in its design, coordination and in writing the manuscript. All authors read and approved the final manuscript.
